# Large driftwood accumulations along arctic coastlines and rivers

**DOI:** 10.1038/s41598-025-17426-y

**Published:** 2025-09-12

**Authors:** Carl Stadie, Martin Brandt, Ingmar Nitze, Xiaoye Tong, Siyu Liu, Ankit Kariryaa, Sizhuo Li, Florian Reiner, Tabea Rettelbach, Guido Grosse

**Affiliations:** 1 Alfred Wegener Institute Helmholtz Centre for Polar and Marine Science, 14473 Potsdam, Germany; 2 Department of Geosciences and Natural Resource Management, University of Copenhagen, Copenhagen, Denmark; 3 Technical University of Denmark, Kongens Lyngby, Denmark; 4 Institute of Geosciences, University of Potsdam, 14469 Potsdam, Germany

**Keywords:** Physical oceanography, Boreal ecology

## Abstract

Driftwood deposits along Arctic coastlines play key ecological roles and serve as indicators of past environmental conditions. Yet, there is a lack of knowledge regarding large-scale distribution patterns, which are important to assess its ecological and geomorphic impacts and carbon stocks. Here, we present a systematic mapping of Arctic driftwood in the North American low Arctic using PlanetScope nano-satellite imagery. We identify 19,717 driftwood deposits covering 22,960,000 m^2^ . Driftwood accumulates in clusters near major river deltas, strongly correlating with boreal forest cover within river catchments. Accumulation declines sharply beyond 200 km from river mouths, challenging current narratives of predominantly long-range redistribution. We compare the performance of driftwood-mapping using PlanetScope imagery with sub-metre aerial imagery. Our method underestimates the total driftwood area by 23.18% but captures large deposits with high accuracy (-4.28% bias). Our assessment highlights the abundance of driftwood on Arctic coastlines and forms a baseline for exploring its temporal variability across large regions, its role in coastal erosion mitigation, and its importance as a carbon sink.

## Introduction

Arctic shores are characterised by extensive deposits of driftwood^1–3^, primarily composed of pine, spruce, and larch logs that originate from boreal forests^4^. These logs are transported by rivers to the ocean, where they are deposited along the coastline^5,6^ as either dispersed individual logs or in large agglomerations, forming berms and mats^7^, some of which cover areas exceeding 11 hectares^2^. Driftwood deposits play a crucial role in linking marine and terrestrial ecosystems^8^. They act as agents of dispersion^9–11^, provide essential habitats and nutrients^8,11,12^, stabilise beach sediments^8,13^, and serve as a vital source of wood for Arctic communities in the otherwise treeless tundra^5,14,15^.

More recently, driftwood has come into focus as an environmental proxy for multiple scientific disciplines. The position and origin of Arctic driftwood deposits have been used to reconstruct historic storm flood events and Arctic sea-currents^16–19^. Its location and sorting provide valuable insights into changing oceanographic conditions^20,21^, while fluctuations in its influx offer important indicators of shifting sea-ice dynamics^22,23^. Additionally, driftwood has proven useful for assessing marine pollutants^24^ and serves as a rich archive for dendrochronological data in the Arctic^1,25,26^. A growing number of studies has also focused on its role in the carbon cycle, suggesting that driftwood acts as a substantial carbon sink^2,26^.

Despite its ecological and scientific importance, most research on driftwood has focused on riverine settings, assessing the formation of wood rafts, logjams, and other structures^7,28,29^, or its impact on infrastructure like bridges, dams, and hydropower stations^30,31^. In contrast, the spatial patterns, size distributions, and accumulation dynamics of coastal driftwood deposits, which differ fundamentally from their riverine counterparts, remain poorly understood^6,32^. This limits our ability to assess the role of driftwood in Arctic coastal ecosystems and its impact on carbon storage, habitat formation, and geomorphologic stability. Although small scale studies covering individual deposits exist^1,2,33^, without a large-scale perspective, it remains unclear whether patterns observed in these localised studies hold true across broader regions and whether findings from plot-scale studies can be reliably extrapolated^6^.

While efforts have been made to map and characterise driftwood using Earth observation imagery in both riverine and coastal settings^3,34,35^, most studies have relied on sub-metre resolution aerial imagery acquired by aircraft or UAVs, and in some cases, sub-metre resolution satellite imagery^2,3,30^. These studies typically cover small study regions and involve the visual identification and manual digitisation of driftwood^7,18,34^. While effective for a small region, this approach is not feasible for mapping driftwood at continental or pan-Arctic scales which is necessary for a meaningful and representative assessment of Arctic driftwood abundance and distribution^3,6,36^.

Recently, the viability of deep and machine learning methods for driftwood mapping was demonstrated^2,3,37^. While these methods can enable rapid large-scale assessments, the restricted availability of suitable imagery has limited the spatial coverage of previous studies. Existing research often argues that imagery of sub-metre spatial resolution is necessary to accurately map driftwood in an automated manner^3,34,37^. Consequently, studies frequently depend on aerial imagery or commercial satellite data with sub-metre resolution like GeoEye, Quickbird, or Worldview^2,3^. However, this reliance introduces temporal inconsistencies, as images are often acquired over extended periods due to the limited coverage of polar regions, sometimes spanning several decades^2^. Such temporal disparities can distort assessments of driftwood dynamics and result in inconsistent mapping of driftwood deposits. Moreover, sub-metre commercial satellite imagery is inherently expensive in terms of acquisition, storage, and processing, further limiting its use for large-scale driftwood mapping. As a result, a critical gap exists in the ability to map driftwood deposits and assess the abundance and distribution of driftwood automatically and on a large scale, severely hindering the progress of the field.

To overcome these limitations, here we take advantage of recent advances in deep learning, particularly in detecting small and sparse features from high-resolution Earth observation imagery,^33,34^ to map and analyse the extent and spatial distribution of driftwood deposits along 11,120 km of the North American Arctic coast using more than 31,000 scenes of 3-m spatial resolution PlanetScope nanosatellite imagery, covering an area of 1.3 million km^2^. Our deep learning-driven effort is the largest Arctic driftwood assessment to date and provides novel insights into driftwood abundance and accumulation patterns across a large region. Additionally, we identified spatial driftwood clusters/hotspots highlighting linkages to riverine source regions, oceanographic transport dynamics, and central depositional characteristics. Since the spatial patterns and characteristics of coastal driftwood deposits, including their size and distribution, are largely unknown, our large-scale dataset allows for the generalisation of abundance, accumulation patterns, and potential distribution drivers —insights that could not be inferred from previous local-scale studies. By overcoming the limitations of localised observations, our broader-scale systematic analysis contributes to a better understanding of Arctic coastal driftwood dynamics and offers valuable data for multiple downstream disciplines.

Our work represents a significant step towards filling the gap in the understanding of Arctic driftwood abundance and distribution. It also highlights the potential of advanced remote sensing techniques combined with deep learning to overcome the limitations of traditional mapping methods, paving the way for more extensive and accurate assessments of driftwood dynamics across the Arctic.

## Results

### Mapping driftwood deposits in the North American low arctic coastal lowlands

To identify spatially stable driftwood deposits, we created annual image composites using high-resolution imagery from the PlanetScope nano satellite constellation for 1.3 million km^2^ of coastal areas of the North American Arctic. We then trained a U-Net deep learning model on approximately 3,200 manually labelled large driftwood patch samples from the PlanetScope composites.

We applied the model across the full study region and identified 19,717 driftwood deposits which remained spatially stable between 2019 and 2023, collectively covering a total area of 22,960,000 m^2^. The average size of these deposits was 1,164 m^2^, with the largest individual deposit located in the western Mackenzie Delta, spanning 147,000 m^2^, the equivalent of 20 football fields.,

To explore year-to-year variations and assess the temporal stability of driftwood deposits, we analysed regions with image coverage from 2019, 2021, and 2023. Within these areas, 5,225 driftwood deposits were detected. Of these, 20.66% were consistently identified across all three years, while 79.34% appeared in two of the three images. Despite this variation in detection frequency, the average size of driftwood deposits remained consistent over time, suggesting limited change in deposit size. Visual inspection showed that most deposits persisted in the same locations across years. However, some remobilisation was observed, particularly within dynamic fluvial environments such as the Mackenzie and Yukon River deltas (Supplementary Fig. 4).

**Fig. 1:**
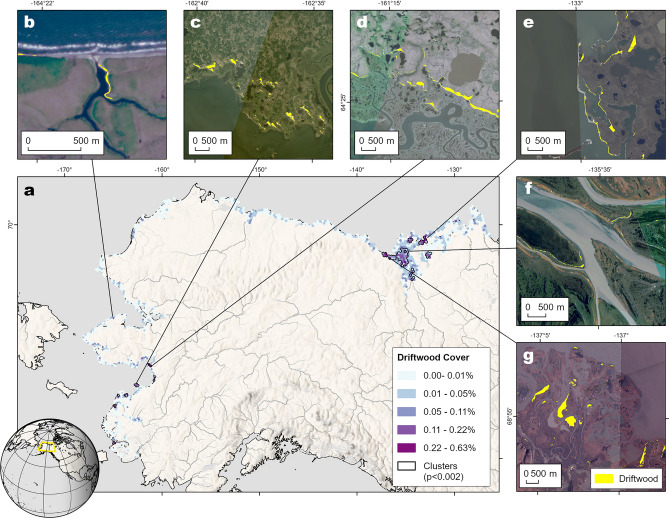
Driftwood cover across the study area.** a** Percentage of driftwood cover aggregated to a 10 × 10 km hexagonal grid with spatial driftwood clusters; **c-g** examples of segmented driftwood deposits overlaid on true colour PlanetScope image composites for: **b** individual deposits on the Cape Espenberg Coast on the northern Seward Peninsula, West Alaska; **c** driftwood mats on Stuarts Island, West Alaska, and **d** Shaktoolik Peninsula north of the Yukon Delta, West Alaska; **e** driftwood mats and coastal berms on the coast east of the Mackenzie Delta, Northwest Canada; **f** berms along islands of the inner Mackenzie Delta, Northwest Canada; **g** the largest detected deposits around Blow River on the western Mackenzie Coastline, Northwest Canada. Variations in colour between images are related to seasonality. More information on the acquisition, processing of PlanetScope composites and licensing is provided under Methods. Detailed views of individual deposits are provided in Supplementary Fig. 5. All maps were produced using ArcGIS Pro version 3.0.1 (Esri Inc., Redlands, CA, USA).

To assess the spatial distribution of driftwood, we aggregated the deposits and their respective areas into a 10 × 10 km grid (Fig. 1a). While driftwood deposits were observed along most coastlines in the study area, deltas of large river systems like the Mackenzie and Yukon rivers and their adjacent coastlines exhibited a particularly high amount of driftwood. This is supported by a moderate to strong degree of spatial autocorrelation (Global Moran’s I = 0.492, p = 0.001). Local hotspots of large driftwood deposits were identified using Anselin Local Moran’s I, revealing ten statistically significant clusters with particularly high amounts of driftwood (p < 0.002). Collectively, these spatial clusters accounted for 7.85 km^2^ of driftwood, corresponding to 34% of the total driftwood area detected by the model.

Key clusters included the Western Mackenzie Delta, particularly along both sides of its West Channel/Shallow Bay. The highest concentration of driftwood in the study area was observed in the Western Mackenzie delta near the mouth of Blow River, with a driftwood cover of 0.63% (Fig. 1g). Other notable clusters in the Mackenzie Delta region included the coastlines west and north of Tuktoyaktuk (Fig. 1e), Richards Island, and the inner delta regions (Fig. 1f). In the Yukon Delta region, Stuart Island (Fig. 1d) and the Shaktoolik Peninsula (Fig. 1c) emerged as major clusters, with the Shaktoolik Peninsula having the highest amount of driftwood in the entire region.

### Characteristics of driftwood deposits

Building on this mapping effort, we analysed the size and spatial distribution of these driftwood deposits to identify key differences between deposits in and outside spatial clusters. Given the ecological and geomorphological significance of driftwood, understanding its spatial patterns and accumulation dynamics at a large scale is crucial. Yet little is known about the spatial variability in deposit size and density which can hint at differences in wood supply or redistribution rates and processes.

Driftwood deposits in spatial clusters were significantly larger than those found elsewhere according to a Mann–Whitney-U-Test (*U* = 35,006,176, *p* < 0.0001), with a larger *U* indicating a larger difference between groups. Outside clusters, deposits were mostly isolated features such as berms or small mats, with a mean area of *x̄* = 1,031 m^2^ (σ = 2,165; Fig. 1b). Deposits inside clusters were larger (*x̄* = 1,551 m^2^, σ = 5,132; Fig. 1c-g), a difference strongly supported by Bayesian analysis (*BF*₁₀ > 100; 95% HDI: [1.1, 1.19]) where the Bayes Factor (BF₁₀) quantifies how much more likely the observed difference is compared to no difference given the observed data. The 95% Highest Density Interval (HDI) represents the most credible range of the group difference. Spatial clusters also exhibited a significantly higher spatial density of deposits (*U* = 7,151, *p* < 0.0001), with an average of 0.85 agglomerations∙km^-2^ compared to 0.16 agglomerations∙km^-2^ outside clusters, though Bayesian evidence for this distinction was weak (*BF*₁₀ > 1; 95% HDI: [1.54, 1.79]).

Further differences emerged based on cluster location. Deposits within deltaic clusters (Fig. 1f) were significantly smaller than those along adjacent coastlines (*U* = 2,191,208, *p* < 0.0001). Deltaic deposits had a mean area of *x̄* = 1,024 m^2^ (σ = 2,075), whereas coastal deposits were substantially larger (*x̄* = 2,670 m^2^, σ = 8,443), with strong Bayesian support (*BF*₁₀ > 100; 95% HDI: [1.1, 1.19]). Despite their smaller size, deposits in deltaic clusters exhibited a significantly higher deposit density (*U* = 973, *p* < 0.01), with a mean of 1.04 agglomerations∙km^-2^ compared to 0.6 agglomerations∙km^-2^ in coastal clusters, though Bayesian analysis provided only weak evidence for this pattern (*BF*₁₀ > 1; 95%, HDI: [1 − 1.2 × 10⁻⁶, 1 + 2.1 × 10⁻⁶]).

**Fig. 2:**
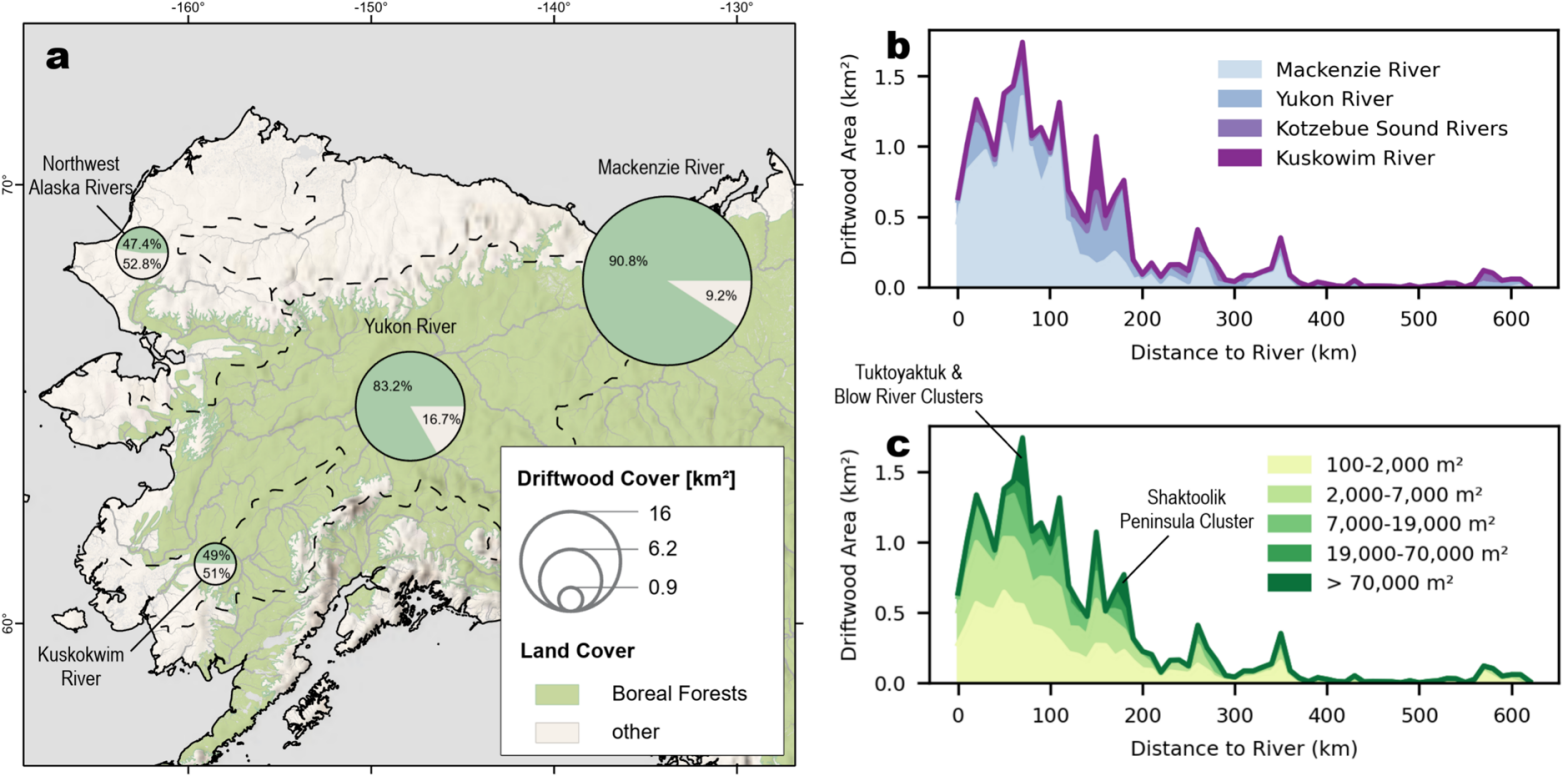
distribution of driftwood by river catchment and distance from river mouth.** a** Coastal driftwood cover by catchment^38^ in relation to catchment forest cover^39^. Only catchments with forest cover were taken into account. Driftwood in catchments without forest cover was assigned to the closest catchment with forest cover along the coastline; **b-c** Driftwood cover by distance from river mouth; **b** grouped by catchment and **c** deposit size. Most driftwood is situated within 200 km of the river mouth and in the Mackenzie River catchment. Deposits larger than 7,000 m^2^ are limited to a 200 km distance from the river mouth. Spikes of large deposits are caused by large deposits in the Mackenzie and Yukon Delta. All maps were produced using ArcGIS Pro version 3.0.1 (Esri Inc., Redlands, CA, USA).

To compare driftwood originating from different Arctic river catchments, we focused on rivers with boreal forests in their catchment areas, as only these systems contribute driftwood to oceans and coastlines. In the study area, boreal forests are restricted to six specific river basins; therefore, our analysis included the Mackenzie and Yukon rivers, which have the highest proportion of forested land within and driftwood cover in their catchments (Fig. 2a), the Kuskokwim River and the Noatak, Kobuk, and Selawik rivers grouped as the "Northwest Alaska rivers" due to the proximity of their mouths. River catchments lacking boreal forest cover were assigned to the nearest forested catchment along the coastline to account for potential driftwood input from forested catchments. This approach ensures that only driftwood originating from boreal forests is considered in the analysis. A strong positive correlation was identified between driftwood-covered area and the proportion of forest cover in the respective catchments (Spearman *ρ* = 0.90, p < 0.05). Bayesian analysis provided robust evidence for this relationship (BF₁₀ > 100; 95%, HDI: [0.8, 0.99]).

While rivers are known as the dominant conveyors of driftwood towards the oceans, its pathways once reaching open waters are still unclear. Thus, we explored the relationship between the cumulative driftwood-covered area and the distance to the nearest river mouth to assess whether driftwood remains near its source rivers or undergoes widespread redistribution. A significant negative correlation was observed (Spearman *ρ* = -0.85, p < 0.0001), indicating that driftwood accumulation decreases with increasing distance from the river mouth. Specifically, 83.2% of total driftwood cover accumulated within a 200 km distance of the river mouth. This relationship was consistently observed across all catchments (Fig. 2b) and across deposits of varying sizes (Fig. 2c). Bayesian analysis strongly supported this finding (BF₁₀ > 100; 95% HDI: [-0.878, -0.696]).

### Evaluation with driftwood estimates from aerial imagery

We used 700 km^2^ of aerial images acquired with a high-resolution camera system during flight campaigns in Alaska and North West Canada in 2019, 2021 and 2023 (for further details refer to methods) to evaluate the PlanetScope based predictions. A separate deep learning segmentation model of similar architecture was trained (sensitivity 92.69% and specificity 92.36%) on the aerial images. While similar in architecture to the PlanetScope-model, the parameterisation differed minimally (Supplementary Table 1). This secondary model served as an independent benchmark to validate the PlanetScope-based predictions and facilitated a comparison between this approach and the conventional use of aerial imagery. Our deep learning model trained to detect large driftwood deposits in PlanetScope images achieved an accuracy of 98.43% (specificity 99.73%, sensitivity 65.59%, IoU = 0.578), respectively.

**Fig. 3:**
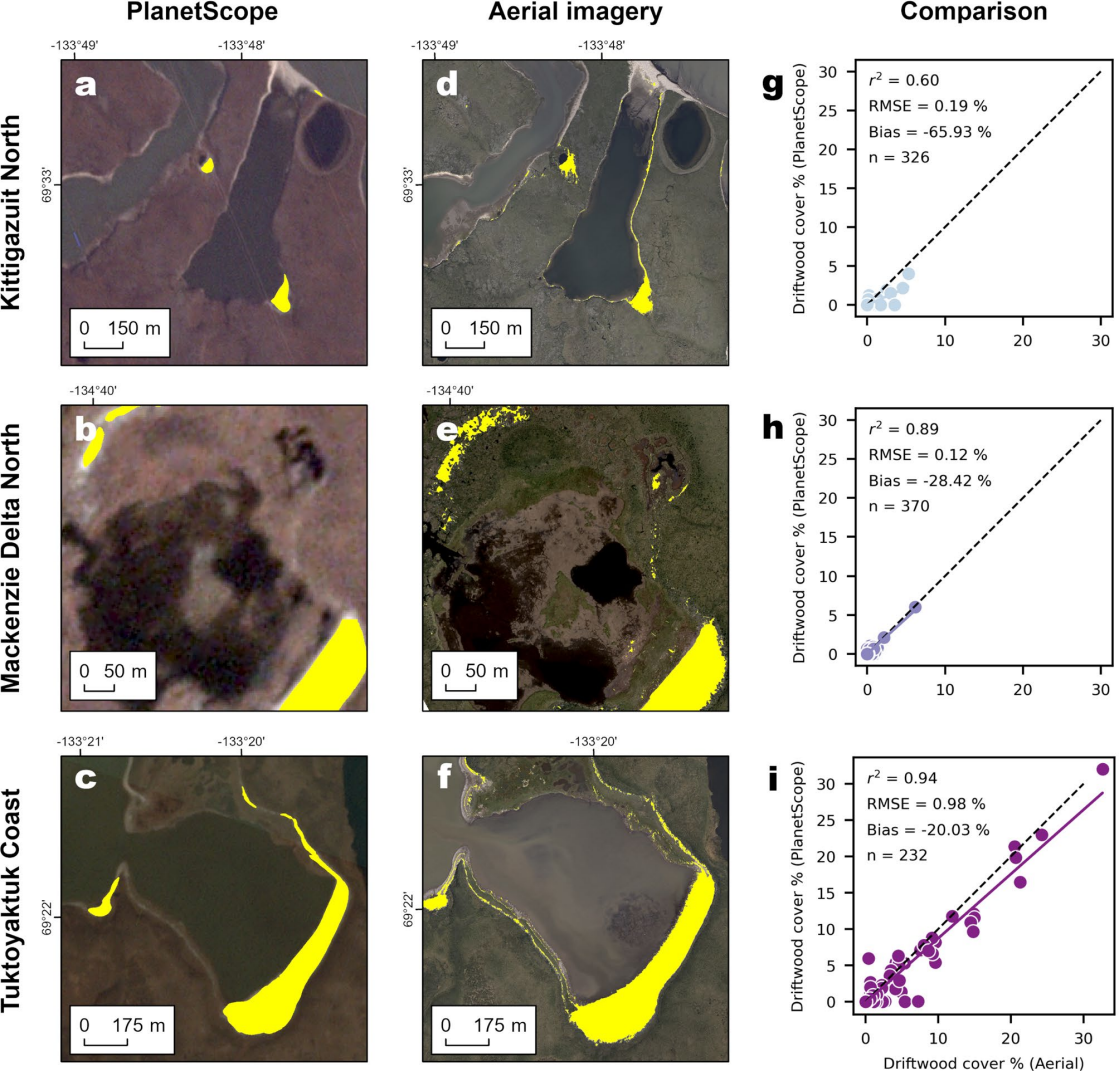
Comparison of PlanetScope predictions with driftwood estimated from aerial images.** a-c** Segmented PlanetScope driftwood deposits on PlanetScope composites; **d-f** Driftwood estimates from the aerial model overlaid on the corresponding images;** g-i** scatterplots of PlanetScope driftwood estimates and driftwood estimates derived from aerial images within 512 × 512 m plots for **g** 326 plots from the northern Mackenzie Delta; **h** 370 plots from Kittigazuit Coast on Richard’s Island in the eastern Mackenzie Delta; **i** 232 plots from the coastline south of Tuktoyaktuk east of the Mackenzie River delta. More information on the acquisition, processing of PlanetScope composites and licensing is provided under Methods. All maps were produced using ArcGIS Pro version 3.0.1 (Esri Inc., Redlands, CA, USA).

The comparison of driftwood cover estimates derived from PlanetScope images and aerial imagery aggregated to a 512 × 512-m grid revealed a very high correlation between the two datasets (*r*^*2*^ = 0.90, slope = 0.86). However, the PlanetScope model missed 23.18% of the total driftwood area identified in the aerial imagery. The majority of this error originated from driftwood deposits with an area smaller than 100 m^2^, which made up 19.75% of driftwood in the aerial estimates. These deposits fell below the model’s minimum mapping unit and were therefore omitted entirely (Supplementary Tab. 2). Consequently, areas dominated by marginal driftwood deposits—consisting mostly individual logs—were poorly represented. These small or overgrown deposits, clearly visible in the aerial imagery (Fig. 3d-f), were often omitted or simplified in the PlanetScope-based predictions (Fig. 3a-c).

In contrast, regions dominated by large driftwood deposits were accurately captured by the PlanetScope model. Deposits larger than 10,000 m^2^ exhibited the smallest error, with their total area being underestimated by 3.97% (Fig. 3g-i). When excluding driftwood deposits smaller than 100 m^2^ from the evaluation—thus focusing only on deposits above the model’s minimum mapping unit—the overall error was reduced to an underestimation bias of 4.28%.

## Discussion

This study presents a systematic, cross-national mapping of driftwood deposits in the North American Arctic, identifying a total of 22,960,000 m^2^ of driftwood across 19,717 deposits, which roughly corresponds to four times the area of New York’s Central Park. Of these 22,960,000 m^2^ 23.28% were contributed by deposits smaller than 100 m^2^. By using high-resolution PlanetScope satellite imagery and deep learning-based image segmentation, this study enables the large-scale mapping of driftwood deposits along Arctic coastlines. Compared to previous research relying on sub-metre aerial or satellite imagery^2,3,16,18^, this approach provides a broader spatial perspective while maintaining high classification accuracy and detail. Our results show that driftwood is not randomly distributed along Arctic coastlines but is concentrated in distinct clusters proximate to the outlets of large river systems, with a clear differentiation between deltaic and coastal deposits. Deposits in deltas tend to be smaller and more densely packed, while coastal deposits are larger and more sparsely distributed. Although the accumulation of driftwood in clusters or hotspots has been previously described, this study provides a data driven quantification and localisation of their extent beyond the plot scale. These results offer a quantification of the number and amount of driftwood and highlight broader coastal accumulation patterns, moving beyond prior studies that focused on riverine driftwood^2,6,29,40^ .

Although the processes governing driftwood accumulation are poorly understood, our results suggest that the depositional setting strongly influences the persistence and morphology of driftwood agglomerations. Coastal deposits typically are extensive driftwood mats^7^ located in embayments or sheltered lagoon-like areas, often pressed against elevated landforms that stabilize them over time. In contrast, deltaic deposits are not just significantly smaller in size, but also predominantly consist of berms along river channels or small, loose mats on floodplains^2^, where they are subject to frequent remobilization by high-water events. Our findings suggest that in dynamic delta environments, driftwood is subject to frequent repositioning due to hydrological fluctuations preventing the formation of large perennial accumulations, whereas in coastal settings, deposits remain more stable and can accumulate over extended periods.

This pattern is consistent with the frequency and scale of hydrological events capable of redistributing driftwood deposits. Along the Beaufort and Chukchi Sea coasts, storm surges capable of displacing driftwood deposits exceed two metres above mean sea level and occur at intervals of 25–50 years, while events exceeding three metres are likely to recur only once every 100 years^17–19^. In the Bering Sea region, including the Yukon Delta and Norton Sound, storm surges are generally lower in magnitude, typically reaching 1–2 m above mean sea level, but occur more frequently1^8,16^. Although these surges can flood low-lying coastal areas, their ability to remobilise large driftwood deposits remains limited due to extensive vegetation entrapment and geomorphic sheltering^7,8^. Within river deltas, high-discharge events associated with snowmelt and ice breakup are capable of mobilising driftwood nearly every year^28,29^. However, the formation of large coastal agglomerations typically requires sequences of such high-flow years, resulting in estimated recurrence intervals of 10–50 years^2,29^.

Outside of clusters, driftwood accumulation is strongly concentrated within 200 km of river mouths, highlighting the role of rivers as primary transport pathways. While Arctic driftwood is known to be redistributed by sea ice and ocean currents, with some logs reaching distant locations such as Greenland and Svalbard^23,41^, our findings indicate that long-range transport is an exception rather than the norm. Instead, most driftwood is deposited relatively close to its riverine source, with a sharp decline beyond 200 km. Accordingly, distant deposits should be viewed as spatial outliers rather than representative of overall driftwood transport patterns. The consistency of this relationship across river systems and deposit sizes further reinforces the dominance of river-driven influx over gradual coastal redistribution.

The observed threshold on long-range driftwood transport likely reflects a combination of physical and oceanographic constraints^20^. Once driftwood exits a river mouth, its movement is influenced by surface currents, wave action, sea ice, and shoreline morphology^20,21^. In nearshore zones, winds and coastal gyres recirculate driftwood back toward the coast, which likely promotes deposition within the first hundreds of kilometres^23^. Beyond 200 km, logs are increasingly exposed to offshore transport, wave-driven fragmentation, or eventual waterlogging and sinking^6,23,24^.

Driftwood cover along Arctic coastlines is strongly correlated with river catchment size and forest cover, confirming that the primary control on driftwood abundance is the availability of woody material within upstream catchments. While this relationship has long been assumed in riverine environments^7,20,28,42^, this study quantifies its impact at a broad spatial scale and in relation to the abundance of coastal driftwood deposits. In the North American Arctic and Subarctic the Yukon and Mackenzie Rivers emerge as the dominant sources of Arctic driftwood, contributing disproportionately to deposits within their deltas and the adjacent coastlines. This strengthens the understanding of the link between terrestrial and marine carbon cycles, emphasizing the role of Arctic rivers in transporting coarse fraction organic material from boreal forests to the coastal environment.

Our findings do not just provide insights into spatial driftwood characteristics, which had previously been largely unknown^6^. They also allow for extrapolation of localized plot-scale observations to broader regions and, beyond providing a large-scale map of driftwood deposits, have broad implications for multiple disciplines. Driftwood has been widely used as a proxy in oceanography^16,18^, geomorphology^7^, and ecology^12^, providing insights into past storm surges, sediment transport, and habitat availability. By quantifying the spatial distribution of driftwood at an unprecedented scale and a high level of detail, this study provides a critical dataset for refining models of Arctic coastal change and improving reconstructions of past environmental conditions. The spatial heterogeneity of driftwood deposits also suggests that they could serve as important data for oceanographic circulation models, particularly those estimating the movement of floating coarse organic material or marine litter.

The use of PlanetScope imagery enabled high-resolution driftwood mapping over a large area, but reliance on commercial satellite data remains a limitation. While the 3 m resolution of PlanetScope is well suited for the detection of extensive driftwood mats, it does not allow the identification of individual logs, which are either not visible in the imagery or lost within mixed pixels. As a result, deposits smaller than 100 m^2^, such as small, uncompacted piles or scattered matrices of isolated logs, are systematically missed. This limitation highlights the trade-offs to be considered in large-scale mapping efforts: While Sentinel-2, a free alternative, could support future monitoring by offering better accessibility, its lower resolution may underestimate total driftwood cover even further. Although large deposits are critical for carbon sequestration and habitat diversity, missing smaller deposits, quantified in this study, could lead to incomplete assessments. Super-resolution techniques^43^ may improve Sentinel-2’s utility by enhancing image sharpness, though they cannot detect driftwood not visible in the original imagery.

Many of these smaller features consist of scattered logs or fragmented wood that fall below the minimum mapping unit, especially in floodplains or dynamic deltaic environments^2,6^. Although they contribute little in terms of mapped area, they still hold ecological value by providing microhabitats^12^, influencing moisture regimes, or contributing to organic fluxes^7,8^. Recognizing the distinction between dense accumulations and sparse, structurally open deposits is therefore important when interpreting their ecological function or transport dynamics.

The problem of underestimating driftwood becomes even clearer when comparing our estimates to similar studies. A study focused on mapping driftwood in the Mackenzie Delta from sub-metre satellite imagery^2^ estimated a driftwood-covered area approximately 50% higher than reported here. Part of this deviation can be attributed to differences in spatial resolution between datasets (0.25–0.5 m vs. 3 m) as revealed by our evaluation with sub-metre aerial image estimates; however, additional factors likely contribute. Our study utilised imagery spanning five years (2019, 2021, and 2023), whereas the reference study incorporated imagery collected over an 11-year period (2009–2021). A longer time frame allows for more driftwood movement, particularly in dynamic environments such as the Mackenzie River Delta, increasing the likelihood of double counting. Unlike our study, which accounts for temporal variation by calculating the probability of driftwood presence from three yearly observations and applying a probability threshold to exclude non-stationary deposits, the reference study did not incorporate an equivalent correction. As a result, methodological differences, image acquisition times, and spatial resolution make direct comparisons between these studies challenging.

Our study provides an essential foundation for expanding research into Arctic driftwood’s role in coastal processes, carbon cycling, and environmental change. Future work should move beyond area-based assessments and integrate volume estimations to better quantify the carbon storage potential of driftwood at a pan-Arctic scale. By combining this large-scale dataset with emerging methods for volume estimation—such as LiDAR-based analysis, 3D modelling, or allometric methods—driftwood carbon stock assessments can be vastly improved. Additionally, integrating this dataset with hydrodynamic and ocean current models will refine our understanding of driftwood transport and deposition patterns, supporting better predictions of Arctic coastal change. The strong correlation between driftwood distribution and boreal forest cover further suggests that this dataset could be leveraged to monitor long-term forest-supply dynamics in response to changing climate conditions, particularly shifts in permafrost stability, riverine erosion and river discharge, or also anthropogenic changes such as forestry practices. As Arctic environments continue to undergo rapid transformations, systematic driftwood mapping can serve as a critical indicator of terrestrial-marine connectivity, aiding research into ecosystem stability, sediment transport, and biogeochemical fluxes at high latitudes.

## Methods

### Overview

Our approach builds on a deep learning model to segment driftwood deposits from annual composites of high-resolution PlanetScope satellite imagery covering the North American Subarctic and Arctic coastline and coastal lowlands between the Kuskokwim River (Alaska) and Horton River (North West Canada). The image data was further supplemented by the ArcticDEM digital elevation model. Additionally, we acquired and used very high-resolution aerial imagery of specific target sites to train a second deep learning model to generate a fine-scale map of driftwood deposits, allowing independent validation and uncertainty estimations for the satellite data-based model as well as a comparison to conventional methods for driftwood mapping^2,3^. The entire processing workflow is illustrated in Supplementary Fig. 1.

### Satellite imagery

For the satellite-based mapping, we used high-resolution imagery from the PlanetScope nano-satellite constellation, which provides 4-band (RGB + NIR) multispectral scenes at 3-m spatial resolution. The scenes were provided through a research license via the Planet API, specifically utilizing the PSScene analytic_sr product bundle, which offers atmospherically corrected surface reflectance data across the blue, green, red, and near-infrared bands^44^. Imagery was collected between July and September of 2019, 2021, or 2023, to minimize snow cover. Furthermore, scenes were filtered to ensure no more than 1% cloud cover. In total, 31,814 scenes were acquired, covering an area of 1.3 million km^2^.

### Mosaicking

To streamline processing, we created annual mosaics^45^ and divided the study area into 1 × 1-degree tiles. For each tile, all PlanetScope scenes from the same year were mosaicked, resulting in three yearly datasets (2019, 2021, and 2023) across the study area, comprising 580 mosaics per year. To minimize colour discrepancies between individual scenes, a histogram matching algorithm was applied^46^. Histogram matching is defined as the transformation of the histograms of input images to match that of a larger reference image, thereby ensuring seamless transitions between scenes^47^. The reference images were composites created from Landsat 7/8^48^ and Sentinel-2^49^ scenes, resampled to Landsat’s 30 × 30 m spatial resolution and harmonized to the spectral resolution of Landsat 7, with cloud and shadow masking applied. The composites were generated by computing the pixel-wise medoid of all scenes^50,51^.

In addition to the four spectral bands, we extended the image data by adding information on elevation derived from the ArcticDEM digital elevation model downloaded through Google Earth Engine and resampled to match the spatial resolution of the PlanetScope mosaics as an additional fifth band^52^.

### Aerial imagery

Aerial imagery was acquired during three flight campaigns with the Alfred Wegener Institute Helmholtz Centre for Polar and Marine Research (AWI) Polar-5 and Polar-6 airplanes over 16 dedicated target sites in Alaska and Northwestern Canada in 2019, 2021, and 2023^53^, using the Modular Aerial Camera System (MACS) developed by the German Aerospace Centre (DLR) in its MACS-Polar18 configuration^54^. Image acquisition targeted areas around Utqiaġvik formerly known as Barrow on the Alaskan North Slope in 2019, surroundings of Kotzebue Sound and the northern Seward Peninsula in northwestern Alaska in 2021, and the Mackenzie Delta and adjacent coasts of the Canadian Northwest Territories and Yukon Territories in 2023. During all years, flights were conducted at altitudes between 500 and 1500 m above ground level, resulting in a spatial resolution of imagery between 7 and 20 cm. This image resolution is comparable to or higher than the spatial resolution of image data used in previous studies^3,34,37^.

The aerial images consisted of four bands covering the red, green, blue, and near-infrared spectra. All images were acquired between June and July to avoid snow cover. A total of about 130,000 images were processed into orthomosaics using structure-from-motion techniques, with each mosaic corresponding to a specific target site^53^. In total, the mosaics cover an area of 700 km^2^, with the extent of the mosaics depicted in Supplementary Fig. 2. The target sites cover a wide array of landscapes including settlements, coastlines and deltas of arctic and boreal catchments of varying size.

### Mapping driftwood with deep learning

For our driftwood mapping task, we chose an image segmentation approach, representing state-of-the-art methods in the field of deep learning^55^. We employed an extended version^56^ of the U-Net architecture^57^, which was originally designed for medical image segmentation but has proven effective for the segmentation of Earth observation imagery^46,56^. The architecture was adapted with batch normalization and Tversky loss^58,59^ as the loss function, which is particularly effective for handling imbalanced classes^46,56^. As driftwood deposits in the study area can range in size from a few square metres to multiple hectares, we further modified the kernels in the encoder part to perform dilated convolutions. Dilated convolutions enlarge the perceptive field of the kernel without increasing the overall number of parameters or number of computations by inserting gaps (dilation rate) between the kernel elements^60^. Here, a dilation rate of 2 was used. The model received all available bands (RGB + NIR + DEM) as input. Training data was created from 2,023 manually delineated rectangular training areas across the imagery covering an area of 534.75 km^2^. Supplementary Fig. 3a provides the spatial extent of the training areas.

Within the training areas, driftwood deposits were manually labelled, resulting in a binary classification differentiating between driftwood and background. Only deposits that clearly showed log-like structures in Google Earth or Bing Maps imagery were labelled as driftwood. This was necessary to accurately distinguish between driftwood and similar looking surfaces like bare sand or rock faces^2,3^. In total, 9,622 deposits were labelled in the PlanetScope imagery. The labelled training areas were rasterized for further processing. The training dataset was bicubically up-sampled from 3 to 1 m spatial resolution to preserve spatial detail and enhance the model’s ability to detect small features^46^. The training images were randomly split into training-, validation- and test sets, with 60% of images being used for training, 20% of images being used for validation during training, and 20% of images being held back as a test set of the final model evaluation.

Training patches of 512 × 512-pixels were randomly sampled from the training areas. To improve model generalization, data augmentation techniques were applied, including vertical and horizontal flipping, random cropping, linear contrast enhancement, piecewise affine transformation, perspective transformation, gamma contrast adjustment, patch level normalization, and Gaussian blur^61^. These augmented patches, along with the original patches, formed the basis for the gradient-based optimization of the U-Net parameters. The training process was monitored by tracking the loss and validation error, with the best-performing hyperparameters selected for the final model. Prediction was carried out on 512 × 512-pixel patches from both the aerial images and PlanetScope mosaics, with a 2% overlap between patches to ensure smooth transitions in the predicted outputs. If driftwood was detected in any of the overlapping pixels, the final classification for that pixel was driftwood.

Although the initial image dataset and corresponding training data covered a large part of the entire North American Arctic, for economic reasons only areas along the coastline and coastal lowlands of the North American low Arctic between the Kuskokwim River (Alaska) and Horton River (Northwest Canada) were targeted for prediction. Since the PlanetScope imagery consisted of mosaics from three different years, each year’s mosaics were processed individually. The binarized prediction confidences from each year were combined into a final output by calculating the probability of driftwood presence for each pixel. A probability threshold of ≥ 0.6 was used to determine the presence of driftwood, while a probability threshold of ≥ 0.5 was used to delineate the shape of the deposit in the final classification, which helped to reduce artifacts, prevent double counting of repositioned deposits and fill gaps in the annual datasets. To avoid any distortions caused by varying spatial resolutions, particularly in the aerial datasets, all predictions were converted from raster to vector format, and the area of each polygon in the resulting dataset was calculated.

To further improve the quality of the prediction, we created a water mask from the DynamicWorld dataset^62^, by assigning the water class to all pixels that were classified as water in more than 20% of the observations in 2019, 2021 and 2023. From this, we derived the water covered area for each potential deposit, and omitted all deposits with water coverage larger than 20% from the prediction. We further omitted deposits intersecting roads and building footprints. Finally, deposits with more than 5 holes (interior rings within polygon geometries) were excluded from the final prediction, to further reduce the number of artifacts. The threshold was defined from visual inspection of the result.

### Spatial analysis of driftwood distribution and statistical validation

To investigate the spatial distribution of driftwood deposits within the study area, we aggregated deposit data into a hexagonal grid with a cell size of 10 × 10 km. This spatial framework allowed a systematic analysis across the region. Clusters or hotspots of driftwood accumulation were identified using Anselin Local Moran’s I statistic^63^, with Queen’s Case^64^ employed as the conceptualization of spatial relationships to account for adjacency effects.

To explore differences in the area and concentration of driftwood deposits within versus outside clusters, as well as between clusters located in river deltas and those on adjacent coasts, we employed the Mann–Whitney U test^65^. This non-parametric test was chosen due to the highly skewed distributions of the analysed variables, ensuring robust comparisons. To confirm the patterns identified by the Mann–Whitney U test and quantify the strength of evidence, we conducted a Bayesian t-test using the BEST (Bayesian Estimation Supersedes the t-test) framework^66^.

Furthermore, we examined the relationship between driftwood cover and boreal forest cover within river catchments. Driftwood in the Arctic tundra originates from southern boreal forests and relies on river systems for transport to the Arctic Ocean and its coastlines^41,42^. Therefore, only river catchments with forest cover were included in the analysis. Catchments without forest cover were assigned to the nearest conforming catchment along the coastline, under the assumption that driftwood from non-forested catchments is more likely to originate from nearby forested areas. Driftwood deposits were aggregated within these refined catchment boundaries.

To investigate the relationship between cumulative driftwood cover and the distance to the nearest river mouth, the distance from each deposit to the mouth of the closest river with a forested catchment was calculated. For river deltas, the mouth of the river was defined as the closest channel outlet. The Spearman rank correlation coefficient (*ρ*)^67^ was used to assess the significance of these relationships. Bayesian approaches^68^ were applied to confirm the observed correlations, providing robust statistical support.

### Evaluation

To evaluate the difference between the use of PlanetScope imagery and conventionally used aerial images we trained a separate model to segment driftwood from the MACS aerial imagery with an architecture similar to the model used on the PlanetScope imagery, receiving all available bands (RGB + NIR) as input. The model was trained on manually labeled training data in 1,017 training areas, totaling 2.5 km^2^. An overview of the training areas is shown in Supplementary Fig. 3 b-d. Within the training areas, 8,444 entities of driftwood were manually labeled. Individual logs were labeled as distinct entities, while continuous clusters like berms or mats were labeled as single entities. The training data was rasterized, but not upsampled, as the spatial resolution of the imagery was deemed sufficient and randomly split into training, validation and test sets.

The model was trained on 512 × 512-pixel wide patches, which were randomly sampled from the training data, with the above-mentioned data augmentation techniques applied. The final prediction was performed on 512 × 512 patches, with the result being converted to vector format to calculate the area of the segmented deposits. The resulting estimate was used to quantify the error introduced by the coarser spatial resolution of the satellite imagery used in the first model and evaluate the use of coarser resolute imagery as compared to the conventionally used sub-metre aerial imagery. Detailed parameter settings for both models are provided in Supplementary Tab. 1.

To evaluate the performance of both models, independent test datasets were used in addition to visual assessments to evaluate the models’ accuracy. The test dataset is independent of the training process, ensuring unbiased model evaluation.

The PlanetScope model’s performance compared to the use of aerial imagery was assessed by comparing its predictions against those from the aerial model. Predictions were aggregated into a 512 × 512 m grid, and the driftwood area within each cell was compared across target sites. We calculated the relative bias between the PlanetScope and aerial-derived driftwood coverage. This analysis facilitated the comparison of high-resolution satellite data with traditionally used very high-resolution aerial imagery^2,3,37^.

### Auxiliary data

We utilised the Dynamic World dataset^62^ to mask water areas in the final predictions. The water mask was created by assigning the water class to all pixels in Dynamic World’s water band where water persisted for more than 20% of observations between July and September in 2019, 2021, and 2023. Data on infrastructure was sourced from OpenStreetMap^69^, while information on catchments was obtained from the pan-Arctic catchment database ARCADE^38^. Non-forested catchments were aggregated to the nearest forested catchment along the coastline. Forest data was derived from Natural Canada’s Extent of the Boreal Forest dataset^39^, and river data was extracted from the HYDRORivers^70^ dataset provided by the HYDROSheds Project.

## Supplementary Information

Supplementary Information.

## Data Availability

The datasets generated during the current study are available from the corresponding author upon request.

## Code availability

The code used to generate the results of the current study are available from the corresponding author upon request.
