# Supplementary material for: Large driftwood accumulations along arctic coastlines and rivers
Source: Sci Rep. 2025 Sep 12;15:32500. doi: 10.1038/s41598-025-17426-y (PMC12432229; doi:10.1038/s41598-025-17426-y)
Supplement: Supplementary file 1 — Supplementary Information. [file 41598_2025_17426_MOESM1_ESM.docx]

**Supplementary Information to:**

**Large driftwood accumulations along Arctic coastlines and rivers**

**
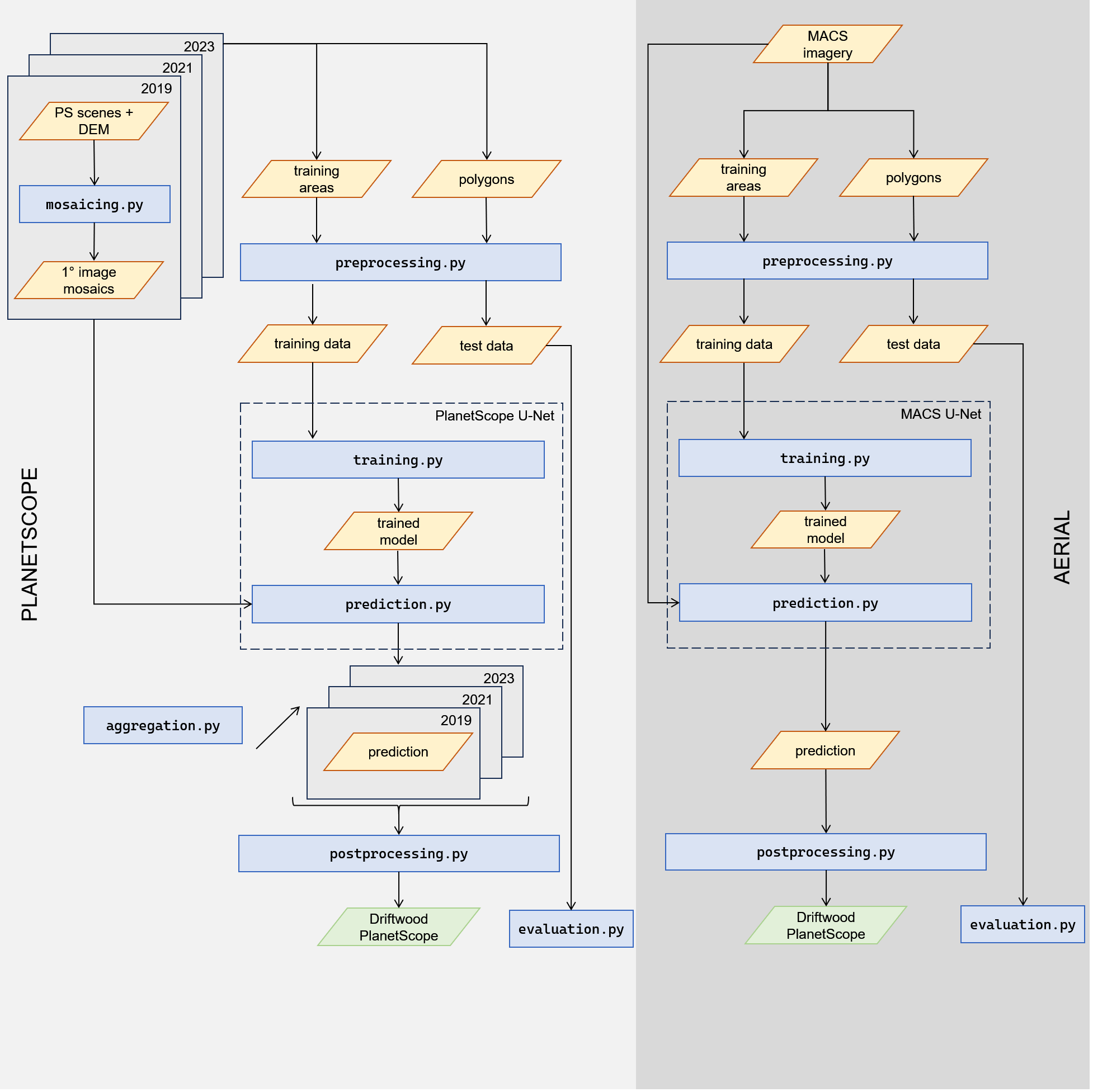
**

***Supplementary Fig. 1: Processing Workflow.*** *We segmented driftwood from PlanetScope mosaics from 2019, 2021 and 2023 using a specifically trained U-Net model. To evaluate the model’s product against conventional approaches for driftwood segmentation, we trained a similar model to segment driftwood from MACS aerial imagery from 16 target sites.*

**
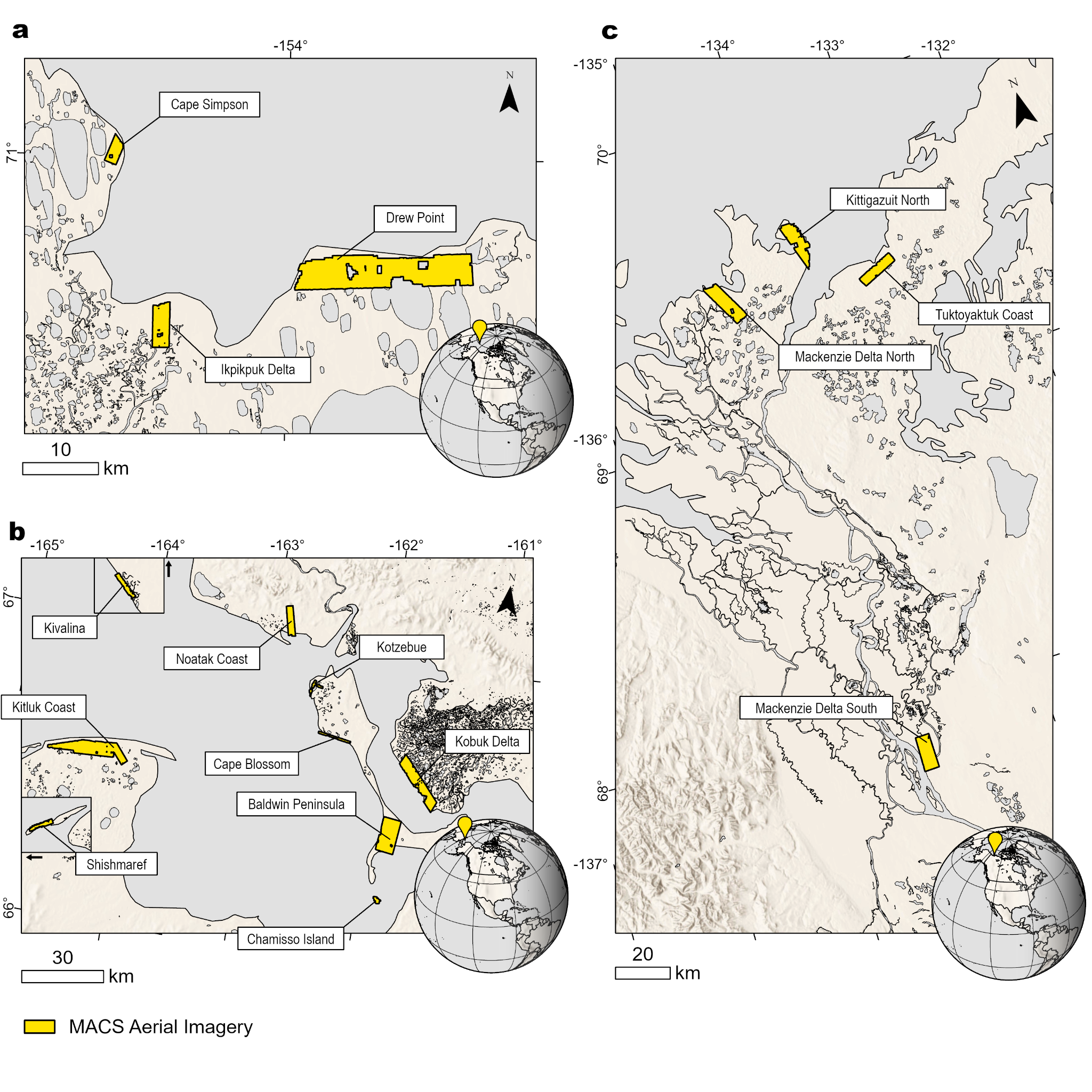
**

***Supplementary Fig. 2: Extent of aerial imagery.***  ***a*** *3 images from 2019 in North Alaska;* ***b*** *9 from 2021 in West Alaska and* ***c*** *4 from 2023 in the Mackenzie Delta covering 700 km² in total were used to evaluate the performance of the PlanetScope model.* All maps were produced using [ArcGIS Pro version 3.0.1](https://www.esri.com/en-us/arcgis/products/arcgis-pro/overview) (Esri Inc., Redlands, CA, USA).

**
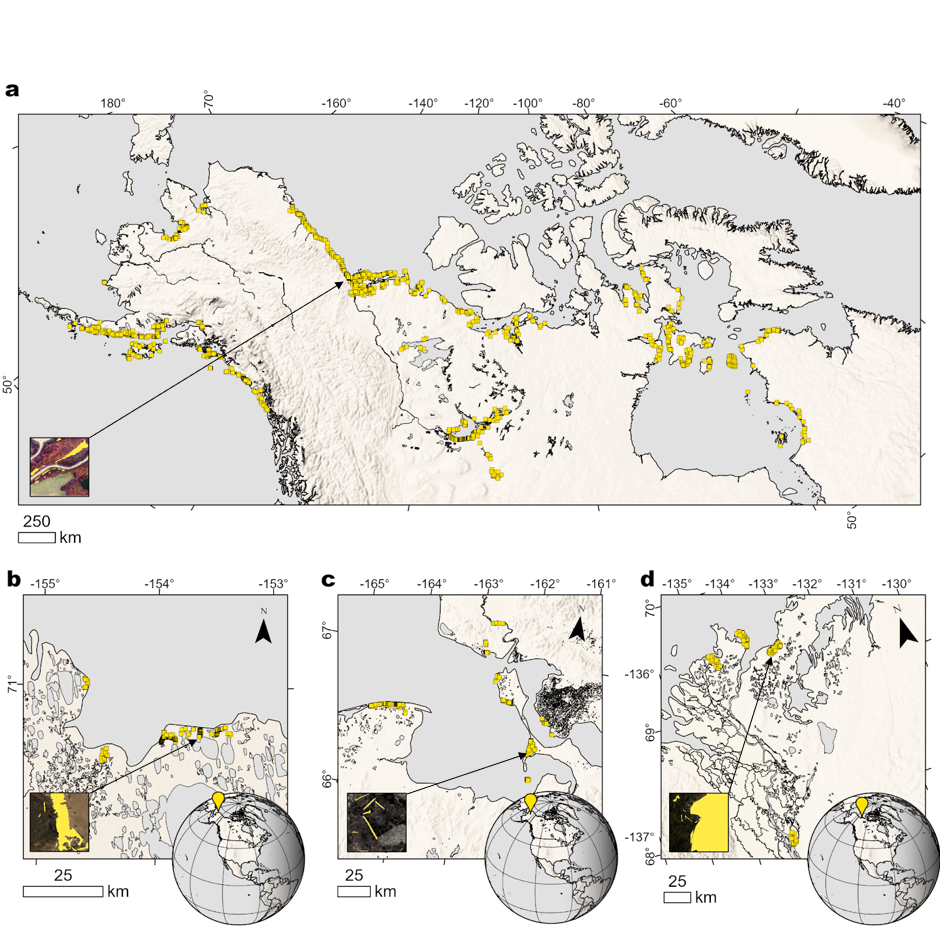
**

***Supplementary Fig. 3: Extent of training areas. a*** *Training areas for the PlanetScope model were laid situated across the entire coastline of the North American Arctic. Training data for the aerial model was collected in the respective images from* ***b*** *North Alaska,* ***c*** *West Alaska,* ***d*** *North West Canada. Within the training areas, driftwood deposits were manually labeled (see inserted images for exemplary training images). All maps were produced using* [*ArcGIS Pro version 3.0.1*](https://www.esri.com/en-us/arcgis/products/arcgis-pro/overview) *(Esri Inc., Redlands, CA, USA).*


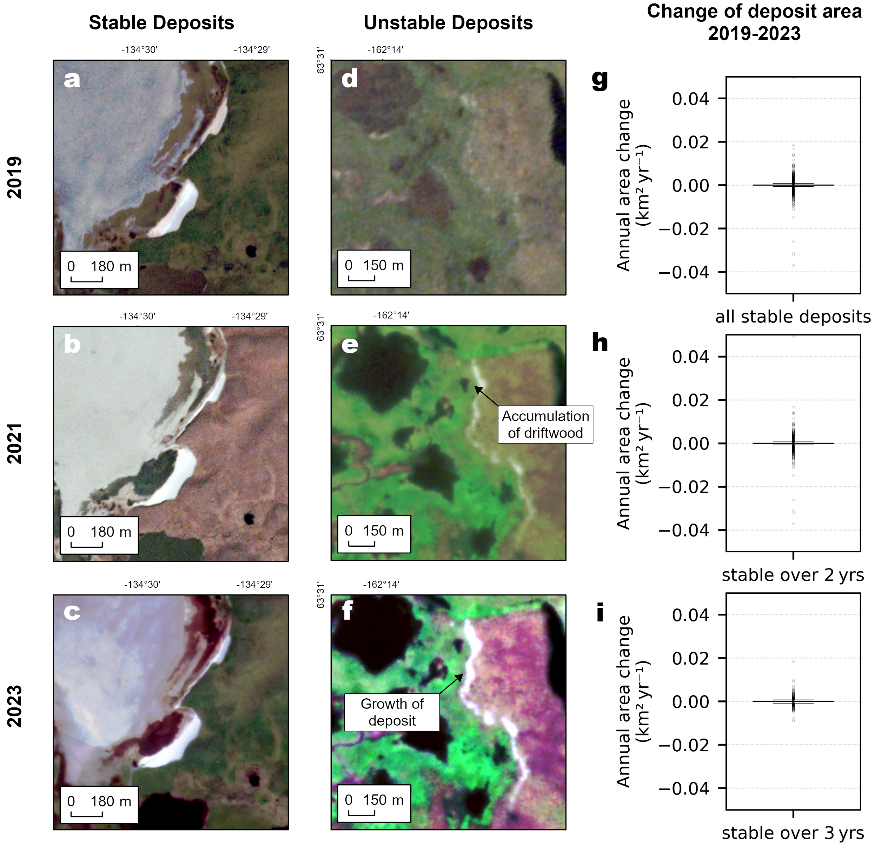


***Supplementary Fig. 4: Examples of temporal dynamics of driftwood deposits. a–c*** *Stable driftwood mat on the Kittigazuit coast, northern Mackenzie Delta, showing no detectable change between 2019, 2021, and 2023. The deposit remains persistent over time, likely due to geomorphic stabilization.* ***d–f*** *Dynamic driftwood berm north of the Yukon River Delta. No driftwood is visible in 2019, but accumulation begins in 2021 and expands by 2023.* ***g–i*** *Annual change in deposit area for subsets of deposits detected in multiple observations:* ***g*** *all deposits observed in at least two consecutive years;* ***h*** *deposits present in exactly two years;* ***i*** *deposits present across all three years. Across all groups, average annual area change is zero, indicating that most deposits remain stable over time, with localized instances of accumulation or remobilization. More information on the acquisition, processing of PlanetScope composites and licensing is provided under Methods. All maps were produced using* [*ArcGIS Pro version 3.0.1*](https://www.esri.com/en-us/arcgis/products/arcgis-pro/overview) *(Esri Inc., Redlands, CA, USA).*

**
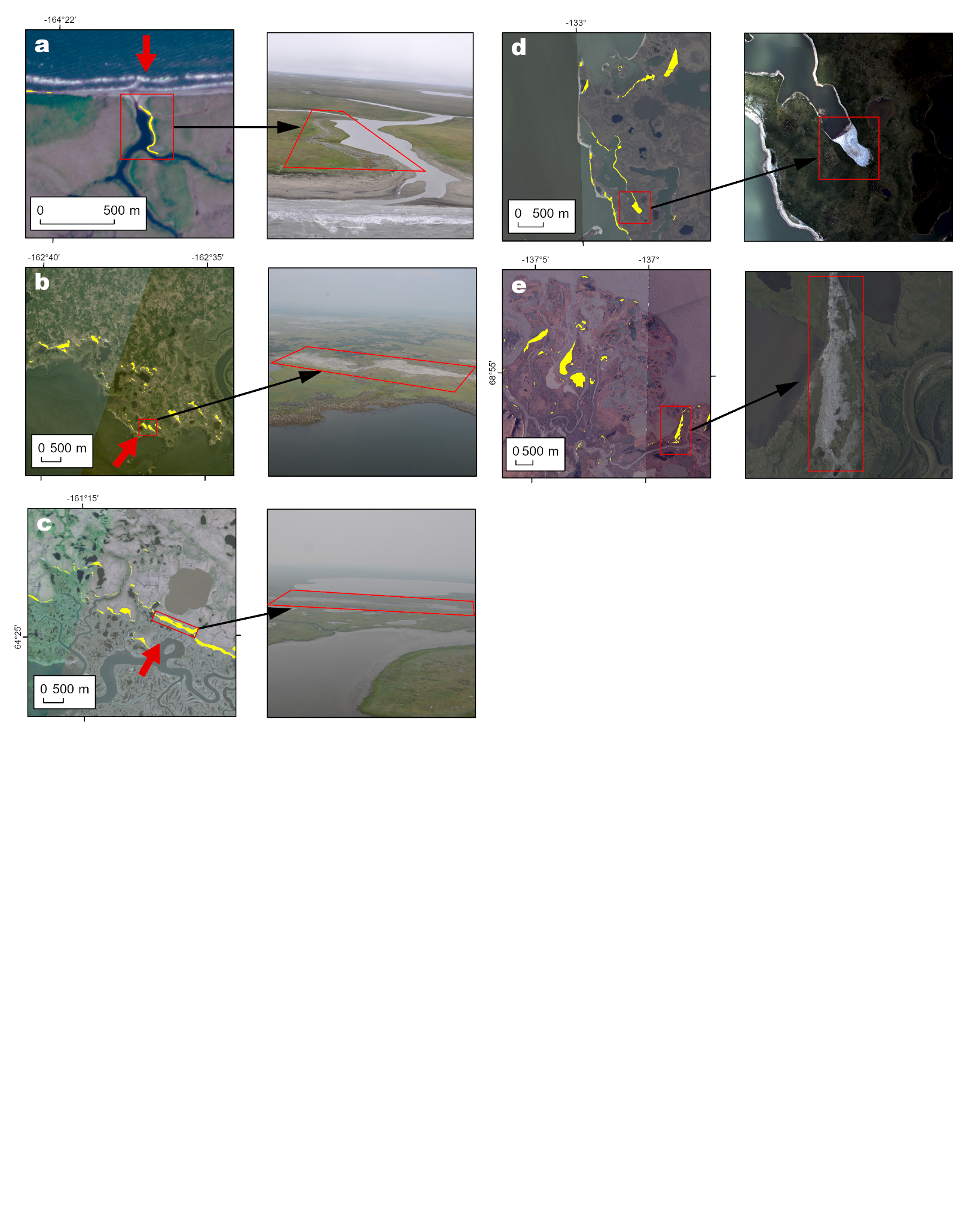
*Supplementary Fig. 5:*** ***Closeup views of selected driftwood deposits. a*** *Driftwood berm on Kitluk Coast on the Northern Seward Peninsula, West Alaska;* ***b*** *Driftwood mats on Stuarts Island, West Alaska;* ***c*** *The 8^th^ largest deposit in Shaktoolik Peninsula north of the Yukon Delta, West Alaska;* ***d*** *Driftwood mats and on the coast east of the Mackenzie Delta, Northwest Canada;* ***e*** *The 15^th^ largest deposit around Whitefish Station on the western Mackenzie Coastline, Northwest Canada. Oblique aerial images from NOAA Shorezone^1^ icensed under CC BY 3.0, aerial orthophotos from AWI MACS flight campaigns^2^. Red arrows indicate the viewpoint of the oblique aerial imagery. More information on the acquisition, processing of PlanetScope composites and licensing is provided under Methods. All maps were produced using* [*ArcGIS Pro version 3.0.1*](https://www.esri.com/en-us/arcgis/products/arcgis-pro/overview) *(Esri Inc., Redlands, CA, USA).*

***Supplementary Table. 1: Parameterisation of deep learning models.***

|  |  | **PlanetScope U-Net** | **Aerial image U-Net** |
| --- | --- | --- | --- |
| **Architecture** | **input size** | 512×512×6 | 512×512×4 |
|  | **channels used** | RGBN+NDVI+DEM | RGBN |
|  | **number of up/downsampling stages** | 5 | 5 |
|  | **kernel size** | 3×3 | 3×3 |
|  | **dilation rate** | 2 | 2 |
|  | **pooling** | 2×2 max pooling | 2×2 max pooling |
|  | **activation function** | ReLU/Sigmoid on Output | ReLU/Sigmoid on Output |
| **Training Parameters** | **loss function** | Tversky loss  ⍺ = 0.7  ꞵ = 0.3 | Tversky loss  ⍺ = 0.5  ꞵ = 0.5 |
|  | **optimizer** | Adadelta | Adadelta |
|  | **learning rate** | 0.001 | 0.001 |
|  | **batch size** | 8 | 8 |
|  | **epochs** | 500 | 500 |
|  | **data augmentation** | horizontal/vertical flip: 50%  cropping: 10%  gaussian blur: 30%  linear contrast: 30%  piecewise affine: 30%  perspective transform: 10% | horizontal/vertical flip: 50%  cropping: 10%  gaussian blur: 30%  linear contrast: 30%  piecewise affine: 30%  perspective transform: 10% |
| **Evaluation** | **dataset split** | training = 60 %  validation = 20 %  test = 20 % | training = 60 %  validation = 20 %  test = 20 % |

***Supplementary Table. 2: Evaluation.***

|  | **deposit area MACS aerial imagery (m²)** | **deposit area PlanetScope satellite imagery (m²)** | **Difference (%)** |
| --- | --- | --- | --- |
| **<100 m²** | 174,544 | 0 | -100 |
| **100-5,000 m²** | 258,260 | 225,747 | -12.56 |
| **5000-10,000 m²** | 88,622 | 105,141 | 18.6 |
| **>10,000m²** | 362,440 | 348,069 | -4.0 |
| **ALL** | 883,866 | 678,957 | -23.2 |
| **ALL > 100m²** | 709,322 | 678,957 | -4.3 |

**References**

1[. NOAA. NOAA ShoreZone Atlas. (2024).](https://www.zotero.org/google-docs/?k4MzXv)

2[. Rettelbach, T. *et al.* Super-high-resolution aerial imagery datasets of permafrost landscapes in Alaska and northwestern Canada. (2023) doi:10.5194/essd-2023-193.](https://www.zotero.org/google-docs/?k4MzXv)
